# Expanding the scope of cyclopropene reporters for the detection of metabolically engineered glycoproteins by Diels–Alder reactions

**DOI:** 10.3762/bjoc.10.232

**Published:** 2014-09-22

**Authors:** Anne-Katrin Späte, Verena F Schart, Julia Häfner, Andrea Niederwieser, Thomas U Mayer, Valentin Wittmann

**Affiliations:** 1 University of Konstanz, Department of Chemistry and Konstanz Research School Chemical Biology (KoRS-CB), Universitätsstraße 10, 78457 Konstanz, Germany; 2 University of Konstanz, Department of Biology and Konstanz Research School Chemical Biology (KoRS-CB), Universitätsstraße 10, 78457 Konstanz, Germany

**Keywords:** bioorthogonal chemistry, carbohydrates, cyclopropenes, inverse-electron-demand Diels-Alder reactions, metabolic oligosaccharide engineering

## Abstract

Monitoring glycoconjugates has been tremendously facilitated by the development of metabolic oligosaccharide engineering. Recently, the inverse-electron-demand Diels–Alder reaction between methylcyclopropene tags and tetrazines has become a popular ligation reaction due to the small size and high reactivity of cyclopropene tags. Attaching the cyclopropene tag to mannosamine via a carbamate linkage has made the reaction even more efficient. Here, we expand the application of cyclopropene tags to *N*-acylgalactosamine and *N*-acylglucosamine derivatives enabling the visualization of mucin-type O-glycoproteins and O-GlcNAcylated proteins through Diels–Alder chemistry. Whereas the previously reported cyclopropene-labeled *N*-acylmannosamine derivative leads to significantly higher fluorescence staining of cell-surface glycoconjugates, the glucosamine derivative gave higher labeling efficiency with protein preparations containing also intracellular proteins.

## Introduction

**Scheme 1:**
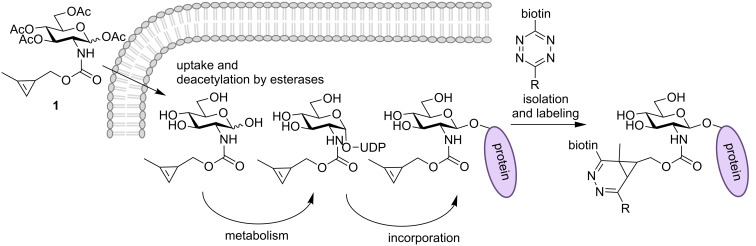
Principle of MOE with Ac_4_GlcNCyoc (**1**) and subsequent ligation by a DAinv reaction: The chemically modified sugar is fed to cells, taken up by the cells and deacetylated by non-specific esterases. The monosaccharide is metabolized and incorporated into glycoproteins (i.e., O-GlcNAcylated proteins). Subsequently, a ligation reaction is performed to visualize the glycan.

The glycan chains of glycoproteins and lipids have been shown to be involved in numerous biological recognition and regulation events [1]. Glycan research, especially the visualization of glycoconjugates in vitro and in vivo, has significantly profited from the recent developments in the area of metabolic oligosaccharide engineering (MOE) and the chemical reporter strategy [2–4]. In this approach, functional groups with a unique reactivity are incorporated into glycoconjugates via the cell’s biosynthetic machinery and are subsequently reacted in bioorthogonal labeling reactions that allow visualization [5–6]. Whereas in the first report on glycan labeling by this approach the ketone–hydrazide ligation was employed [7], later investigations mainly relied on the Staudinger ligation [8] and azide–alkyne [3 + 2] cycloaddition (copper-catalyzed [9–10] or strain-promoted [11–12]). Since the initial reports from 2008 [13–15], more and more laboratories successfully employ the inverse-electron-demand Diels–Alder (DAinv) reaction as a bioorthogonal ligation reaction for different applications [16–18]. In the meantime, the DAinv reaction has also found application in MOE, and several dienophiles, such as terminal alkenes [19], isonitriles [20–21], and cyclopropenes [22–24], have been incorporated in carbohydrate derivatives and detected by reaction with 1,2,4,5-tetrazines [25] (Scheme 1). An important advantage of the DAinv reaction is the fact that it can be orthogonal to the azide–alkyne cycloaddition [22,26–27] which allows dual labeling of two different sugars within one experiment [19,21,23–24].

**Figure 1:**
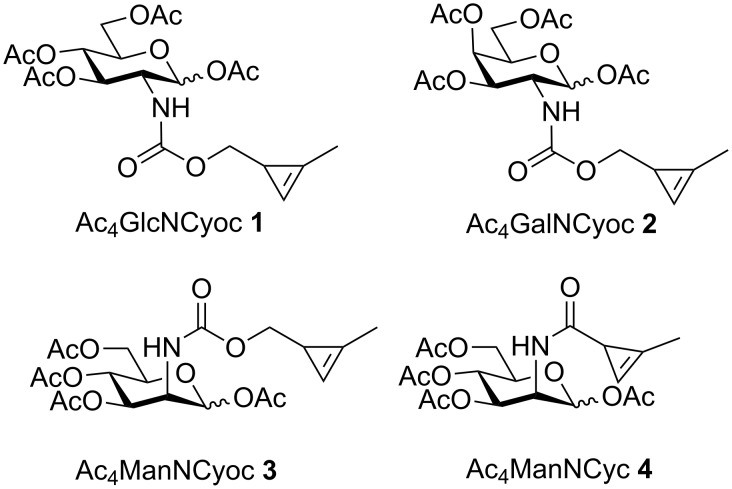
Hexosamine derivatives with cyclopropene tags. Cyoc = (2-methylcycloprop-2-en-1-yl)methoxycarbonyl, Cyc = 2-methylcycloprop-2-ene-1-carbonyl.

Among the dienophiles mentioned above, strained cyclopropenes have the highest reaction rates for DAinv reactions with tetrazines and are small enough to be accepted by cellular enzymes during MOE [22–24]. Also, they are stable in aqueous solution in the presence of biological nucleophiles [22,28]. Consequently, cyclopropene tags were attached by an amide linkage to sialic acid [22] and ManNAc derivatives including Ac_4_ManNCyc (**4**) [23] (Figure 1) to label sialic acid residues on the surface of living cells via MOE. Since carbamate-linked methylcyclopropenes have significantly higher reaction rates in DAinv reactions with tetrazines [22,28], we recently introduced Ac_4_ManNCyoc (**3**) as a derivative for rapid labeling of metabolically engineered cell-surface sialic acids [24]. The application of **3** was prompted by the previous observation that carbamate-modified ManNAc derivatives are also accepted in the biosynthetic pathway [19,29]. Derivative **3** in combination with a sulfo-Cy3-tetrazine conjugate enabled dual sugar labeling by simultaneous DAinv reaction and strain-promoted azide–alkyne cycloaddition in a single step [24]. The potential of Ac_4_ManNCyoc (**3**) for labeling of sialoglycoconjugates was also recognized by others [30]. Sialic acids are prominently positioned at the outer end of membrane glycoproteins which makes them well-accessible for labeling reactions [31]. However it has become of increasing interest to also investigate intracellular glycoproteins. We, thus, developed the glucosamine and galactosamine derivatives Ac_4_GlcNCyoc (**1**) and Ac_4_GalNCyoc (**2**) which are expected to be incorporated into O-GlcNAcylated proteins and mucin-type *O*-glycans [30]. Here, we show that **1** and **2** can be employed for both labeling of cell-surface glycoconjugates (detected by confocal fluorescence microscopy) and isolated glycoproteins (detected by Western blot).

## Results and Discussion

**Scheme 2:**
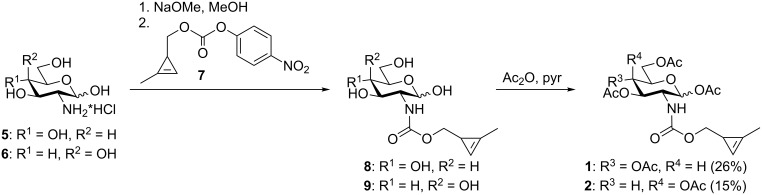
Synthesis of the cyclopropene-modified hexosamine derivatives **1** and **2**.

For the synthesis of the cyclopropene-tagged sugars **1** and **2** we neutralized the corresponding hexosamine hydrochlorides **5** and **6** with sodium methoxide and coupled them to the activated cyclopropene **7** (Scheme 2), the synthesis of which we reported previously [24]. Subsequent acetylation of the carbamates **8** and **9** gave Ac_4_GlcNCyoc (**1**) and Ac_4_GalNCyoc (**2**).

**Scheme 3:**
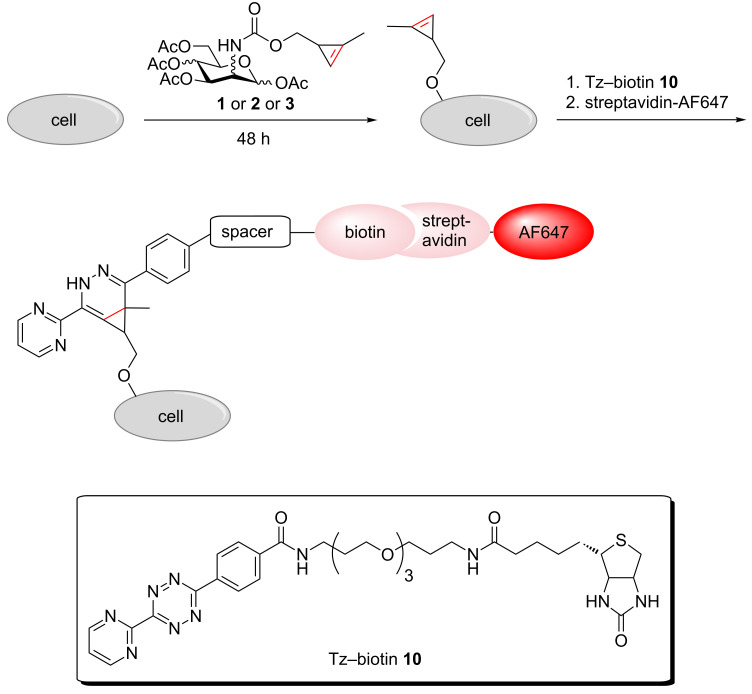
Labeling strategy for metabolically incorporated monosaccharides.

**Figure 2:**
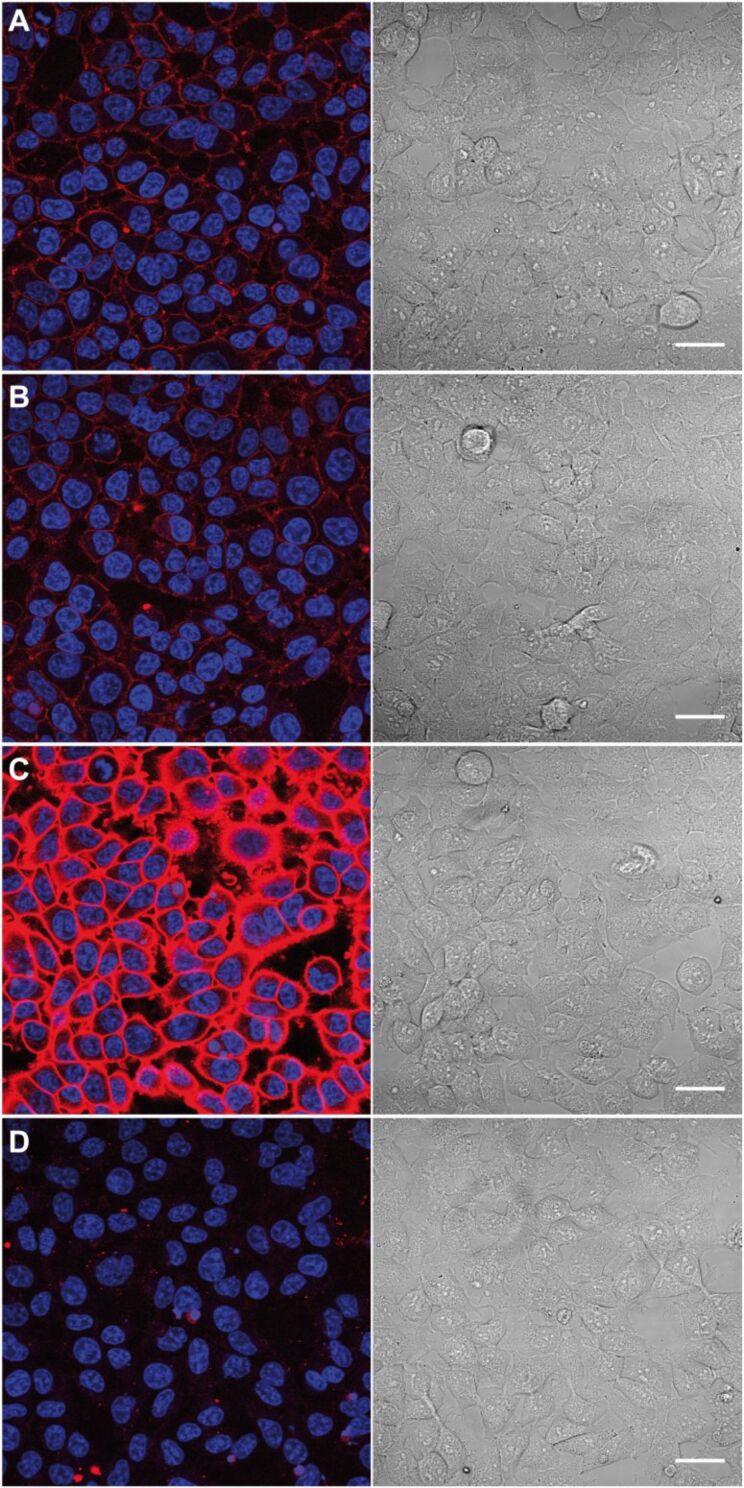
Labeling of metabolically engineered cell-surface glycoconjugates. HEK 293T cells were grown for 48 h with 50 µM Ac_4_GlcNCyoc (**1**, A), 50 µM Ac_4_GalNCyoc (**2**, B), 50 µM Ac_4_ManNCyoc (**3**, C), or with PBS (solvent control, D) and subsequently incubated with Tz–biotin **10** (1 mM, 1 h, 37 °C) followed by incubation with streptavidin–AF647. Nuclei were stained with Hoechst33342. Scale bar: 30 µm.

With the cyclopropene-modified hexosamines in hand we first investigated their metabolic incorporation into cell-surface glycoconjugates of HEK 293T cells. The cells were incubated for 48 h with **1**, **2**, **3**, or solvent control (phosphate buffered saline, PBS) and then reacted with Tz–biotin **10** [19], followed by labeling with streptavidin–AlexaFluor647 (streptavidin–AF647) (Scheme 3). With all three sugars staining of the plasma membrane was detected by confocal laser scanning microscopy of living cells (Figure 2A, B, C). Only the solvent control did not show any membrane staining (Figure 2D, for additional experiments see Figure S1, Supporting Information File 1). Brightfield images were recorded to check the cell morphology. These experiments show that all three cyclopropene derivatives **1**, **2**, and **3** are accepted by the cell’s biosynthetic machinery. However, membrane staining resulting from metabolized Ac_4_ManNCyoc (**3**) was significantly more intense than staining after cultivation with Ac_4_GlcNCyoc (**1**) or Ac_4_GalNCyoc (**2**). Similar experiments were carried out with HeLa S3 cells (Figure S2, Supporting Information File 1). Again, Ac_4_ManNCyoc (**3**) gave the most intensive and Ac_4_GlcNCyoc (**1**) only weak staining. The staining intensity resulting from the galactosamine derivative **2** was in between. Previous work from Bertozzi and coworkers suggests that GlcNAc derivatives such as *N*-azidoacetylglucosamine (GlcNAz) can only enter cell-surface glycans via less efficient conversion of GlcNAz to *N*-azidoacetylmannosamine (ManNAz) and subsequently to the corresponding sialic acid [32–33] following a metabolic pathway known also for the natural sugars [34]. Also, the efficiency by which non-natural GlcNAc and GalNAc derivatives are metabolized is dependent on the type of modification and the cell line. These findings might provide an explanation for the reduced staining intensities obtained with sugars **1** and **2**.

**Figure 3:**
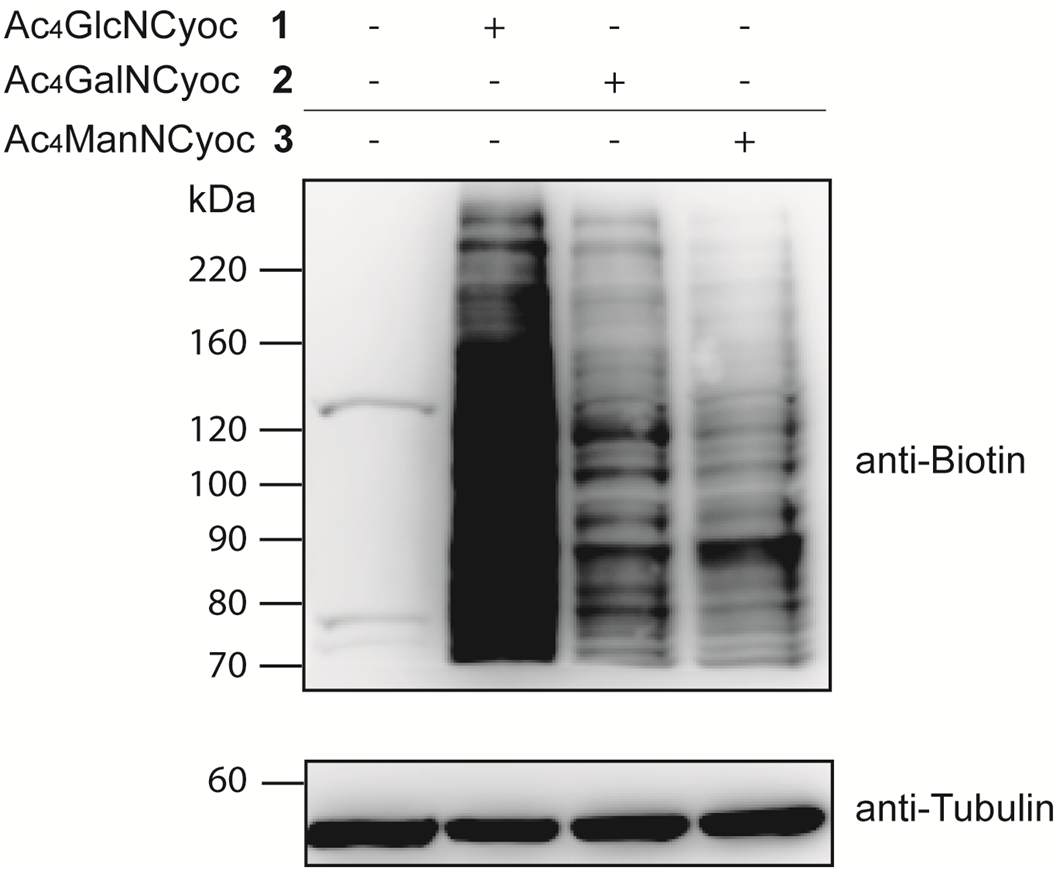
Western blot analysis of soluble glycoproteins. HeLa S3 cells were grown for 48 h with 100 µM cyclopropene-labeled sugar (Ac_4_GlcNCyoc (**1**), Ac_4_GalNCyoc (**2**), or Ac_4_ManNCyoc (**3**)) or with PBS (solvent control), lysed, and the cleared lysate was reacted with Tz–biotin **10** (150 µM, 90 min, rt). Proteins were immunoblotted for biotin and tubulin (loading control). Protein bands visible in the first lane can be explained either by non-specific binding of the anti-biotin antibody or by the occurrence of naturally biotinylated proteins.

We also performed a Western blot analysis of proteins isolated from HeLa S3 cells that had been cultured in the presence of cyclopropene-labeled hexosamines **1**, **2**, or **3**, or with PBS (solvent control). Cells were harvested, lysed and the lysate was cleared by centrifugation resulting in a mixture of intracellular and membrane proteins. In the cleared lysate we performed a DAinv reaction with Tz–biotin **10**. Visualization of glycoproteins was achieved by immunoblotting for biotin, and equal protein loading was verified by blotting against tubulin (Figure 3). All three investigated sugars resulted in labeled protein bands. In this case, samples from cells treated with Ac_4_GlcNCyoc (**1**) produced a significantly higher signal compared to cells treated with Ac_4_GalNCyoc (**2**) or Ac_4_ManNCyoc (**3**). Similar trends were also observed with Jurkat cells by Prescher and coworkers [30]. Since O-GlcNAcylation is a modification primarily found for cytosolic and nuclear proteins [35] and the sample preparation includes the fraction of intracellular proteins, these results suggest that Ac_4_GlcNCyoc (**1**) is suitable to target O-GlcNAcylated proteins.

## Conclusion

In summary, we have shown that cyclopropene-labeled hexosamine derivatives Ac_4_GlcNCyoc (**1**) and Ac_4_GalNCyoc (**2**) can be used to monitor glycosylation of both cell-surface glycoconjugates and isolated, soluble glycoproteins. Whereas Ac_4_ManNCyoc (**3**) leads to significantly higher fluorescence staining of cell-surface glycoconjugates, Ac_4_GlcNCyoc (**1**) gave higher labeling efficiency with protein preparations containing also intracellular proteins, possibly by targeting O-GlcN-acylated proteins. Since O-GlcN-acylation of proteins is associated with numerous crucial biological events, **1** represents a promising probe for future glycomics studies. Of special interest is the fact that cyclopropene tags can be combined with azide–alkyne cycloaddition to achieve dual labeling of two different (sugar) moieties as was shown earlier [19,21,23–24,30].

## Experimental

**General methods.** All chemicals were purchased from Aldrich, Fluka, Dextra, and Carbosynth and used without further purification. AlexaFluor-labeled streptavidin and Hoechst33342 were purchased from Invitrogen. Technical solvents were distilled prior to use. All reactions were carried out in dry solvents. Reactions were monitored by TLC on silica gel 60 F254 (Merck) with detection by UV light (λ = 254 nm). Additionally, acidic ethanolic *p-*anisaldehyde solution or basic KMnO_4_ solution followed by gentle heating was used for visualization. Preparative flash column chromatography (FC) was performed with an MPLC-Reveleris system from Grace. Nuclear magnetic resonance (NMR) spectra were recorded at room temperature on Avance III 400 and Avance III 600 instruments from Bruker. Chemical shifts are reported relative to solvent signals (CDCl_3_: δ_H_ = 7.26 ppm, δ_C_ = 77.16 ppm). Signals were assigned by first-order analysis and, when feasible, assignments were supported by two-dimensional ^1^H,^1^H and ^1^H,^13^C correlation spectroscopy (COSY, HMBC and HSQC). HRMS mass spectra were obtained with a micrOTOF II instrument from Bruker Daltonics. Semi-preparative high performance liquid chromatography (HPLC) was conducted on a LC-20A prominence system (pumps LC-20AT, auto sampler SIL-20A, column oven CTO-20AC, diode array detector SPD-M20A, ELSD-LT II detector, controller CBM-20A and software LC-solution) from Shimadzu under the following conditions. Column: Kinetex 5U C18 100A Axia from Phenomenex (250 × 21.2 mm); flow: 9 mL min^–1^; mobile phase: gradient of acetonitrile with 0.1% formic acid (solvent A) in water with 0.1% formic acid (solvent B). Microscopy was performed using a point laser scanning confocal microscope Zeiss LSM 510 Meta equipped with a Meta detector for spectral imaging.

The synthesis of **1** and **2** was carried out as described for the synthesis of Ac_4_ManNCyoc (**3**) [24].

**1,3,4,6-Tetra-*O*-acetyl-2-deoxy-2-((2-methylcycloprop-2-en-1-yl)methoxycarbonylamino)-D-glucopyranose (Ac_4_GlcNCyoc, 1)**. To a solution of glucosamine hydrochloride (**5,** 2 g, 9.2 mmol) in MeOH (20 mL) NaOMe (18 mL of a 0.5 M solution in MeOH, 9.2 mmol) was added under nitrogen. After stirring for 90 min at room temperature, the solution was added to activated cyclopropene **7** (2 g, 9.7 mmol). After stirring for 48 h at room temperature the solvent was evaporated under reduced pressure. The residue was dissolved in pyridine (20 mL) and acetic anhydride (10 mL) was added. After stirring for 24 h at room temperature additional 5 mL acetic anhydride were added to complete the acetylation. After additional 24 h the solvents were evaporated under reduced pressure. The residue was dissolved in CH_2_Cl_2_ (250 mL), washed with 10 % aq KHSO_4_ (200 mL), satd aq NaHCO_3_ (200 mL) and brine (200 mL). The organic layer was dried (MgSO_4_) and the solvent was evaporated under reduced pressure. The residue was purified by FC (silica, petroleum ether/ethyl acetate 10:1) to afford Ac_4_GlcNCyoc (**1**) as a mixture of anomers (1.08 g, 2.36 mmol, 26%). *R*_f_ = 0.36 (petroleum ether/ethyl acetate 1:1); HRMS *m/z*: [M + Na]^+^ calcd for C_20_H_27_NO_11_, 480.14763; found, 480.14511.

Further purification by semi-preparative HPLC (45% A for 10 min, then 45–70% A for 15 min) allowed separation of the anomers. Retention time β-anomer: 15 min, α-anomer: 17.2 min.

α-Anomer: ^1^H NMR (400 MHz, CDCl_3_) δ 6.53 (s, 1H, =CH), 6.19 (d, *J* = 3.5 Hz, 1H, H-1), 5.31–5.12 (m, 2H, H-3, H-4), 4.75 (d, *J* = 9.1 Hz, 1H, NH), 4.25 (dd, *J* = 12.3, 3.1 Hz, 1H, H-6a), 4.18 (td, *J* = 10.2, 3.3 Hz, 1H, H-2), 4.05 (dd, *J* = 12.5, 2.0 Hz, 1H, H-6b ), 4.02–3.96 (m, 1H, H-5), 3.96–3.85 (m, 2H, CH_2_), 2.18 (s, 3H, OAc), 2.10 (s, 3H, CH_3_), 2.07 (s, 3H, OAc), 2.04 (s, 3H, OAc), 2.03 (s, 3H, OAc), 1.59 (s, 1H, C*H*CH_2_) ppm; ^13^C NMR (101 MHz, CDCl_3_) δ 171.4 (C=O), 170.8 (C=O), 169.3 (C=O), 168.8 (C=O), 156.1 (HNC=O), 120.5, 102.1, 102.0, 91.0 (C-1), 73.1, 73.0, 70.8 (C-3 or C-5), 69.8 (C-3 or C-5), 67.8 (C-4), 61.7 (C-6), 52.9(C-2), 21.0 (OAc), 20.8 (OAc), 20.7 (OAc), 17.22 (CH_2_*C*H), 17.18 (CH_2_*C*H), 11.70 (=C*C*H_3_) ppm.

β-Anomer: ^1^H NMR (400 MHz, CDCl_3_) δ 6.53 (s, 1H, =CH), 5.70 (dd, *J* = 8.7, 1H, H-1), 5.22–5.15 (m, 1H, H-3), 5.11 (t, *J* = 9.5, 9.5 Hz, 1H, H-4), 4.73 (d, *J* = 8.1 Hz, 1H, NH), 4.28 (dd, *J* = 12.4, 4.6 Hz, 1H, H-6a), 4.11 (dd, *J* = 12.4, 2.1 Hz, 1H, H-6b), 3.97–3.87 (m, 3H, H-2, CH_2_), 3.81 (ddd, *J* = 9.8, 4.6, 2.2 Hz, 1H, H-5), 2.12 (s, 6H, CH_3_, OAc), 2.08 (s, 3H, OAc), 2.04 (s, 3H, OAc), 2.03 (s, 3H, OAc), 1.61 (s, 1H, C*H*CH_2_) ppm; ^13^C NMR (101 MHz, CDCl_3_) δ 170.8 (C=O), 169.5 (C=O), 156.3 (HNC=O), 120.64, 120.58, 102.10, 102.07, 92.8 (C-1), 76.8 (C-3 or C-5 or CH_2_), 73.0 (C-3 or C-5 or CH_2_), 72.5 (C-3 or C-5 or CH_2_), 68.1 (C-4), 61.8 (C-6), 55.0 (C-2), 21.0 (OAc), 20.9 (OAc), 20.8 (OAc), 20.7 (OAc), 17.2 (CH_2_*C*H), 11.74 (=C*C*H_3_), 11.73 (=C*C*H_3_) ppm.

**1,3,4,6-Tetra-*O*-acetyl-2-deoxy-2-((2-methylcycloprop-2-en-1-yl)methoxycarbonylamino)-D-galactopyranose (Ac_4_GalNCyoc 2)**. To a solution of galactosamine hydrochloride (**6**, 1.75 g, 8.11 mmol) in MeOH (18 mL) NaOMe (16 mL of a 0.5 M solution in MeOH, 8.06 mmol) was added under nitrogen. After stirring for 90 min at room temperature, the solution was added to activated cyclopropene **7** (1.75 g, 8.4 mmol). After stirring at room temperature overnight the solvent was evaporated under reduced pressure. The residue was dissolved in pyridine (18 mL) and acetic anhydride (9 mL) was added. After stirring for 40 h at room temperature the solvents were evaporated under reduced pressure. The residue was dissolved in CH_2_Cl_2_ (230 mL), washed with 10% aq KHSO_4_ (100 mL), satd aq NaHCO_3_ (180 mL) and brine (180 mL). The organic layer was dried (MgSO_4_) and the solvent was evaporated under reduced pressure. The residue was purified by FC (silica, petroleum ether/ethyl acetate 1:1 –> 1:2) to afford Ac_4_GalNCyoc (**2**) as a mixture of anomers (551 mg, 1.2 mmol, 15%). *R*_f_ = 0.33 (petroleum ether/ethyl acetate 1:1); HRMS *m/z*: [M + Na]^+^ calcd for C_20_H_27_NO_11_, 480.14763; found, 480.14551.

Further purification by HPLC (45% A for 10 min, then 45–70% A for 15 min) allowed purification of the β-anomer. Retention time β-anomer: 15 min, α-anomer: 16.5 min.

β-Anomer: ^1^H NMR (400 MHz, CDCl_3_) δ 6.54 (s, 1H, =CH), 5.72 (d, *J* = 8.7 Hz, 1H, H-1), 5.39 (dd, *J* = 3.6, 1.1 Hz, 1H, H-4), 5.10 (ddq, *J* = 11.4, 3.4, 1.4 Hz, 1H, H-3), 4.61–4.48 (m, 1H, NH), 4.14 (qd, *J* = 11.3, 6.6 Hz, 3H, H-2, H-6 ), 4.02 (td, *J* = 6.5, 1.0 Hz, 1H, H-5), 3.93 (d, *J* = 5.2 Hz, 2H, CH_2_), 2.16 (s, 3H, OAc), 2.14 (s, 3H, OAc), 2.12 (s, 3H, CH_3_), 2.04 (s, 3H, OAc), 2.02 (s, 3H, OAc), 1.61 (d, *J* = 5.6 Hz, 1H, C*H*CH_2_) ppm; ^13^C NMR (151 MHz, CDCl_3_) δ (ppm) 170.5 (2 C=O), 170.3 (C=O), 169.5 (C=O), 156.4 (HNC=O), 120.64, 120.55, 102.14, 102.07, 93.1 (C-1), 73.0 (CH_2_), 71.9 (C-5), 70.4 (C-3), 66.6 (C-4), 61.4 (C-6), 51.5 (C-2), 21.0 (OAc), 20.9 (OAc), 20.8 (OAc), 20.8 (OAc), 17.21 (CH_2_*C*H), 17.23 (CH_2_*C*H), 11.8 (=C*C*H_3_) ppm.

**Cell growth conditions.** As described in [24] HEK 293T and HeLa S3 cells were grown in Dulbecco’s Modified Eagle’s Medium (DMEM) supplemented with 5% FBS, 100 units mL^–1^ penicillin and 100 μg mL^–1^ streptomycin. All cells were incubated in a 5% carbon dioxide, water saturated incubator at 37 °C.

**Fluorescence microscopy with Tz–biotin 10.** HEK 293T cells (22,000 cells/cm^2^) were seeded in 4-well ibiTreat μ-Slides (ibidi) coated with fibronectin (25 µg mL^−1^) and poly-L-lysine (0.01%, 1 h, 37 °C). After 12 h cells were incubated for 48 h with 50 µM cyclopropene-labeled sugar (Ac_4_GlcNCyoc (**1**), Ac_4_GalNCyoc (**2**), or Ac_4_ManNCyoc (**3**)). The sugars were prepared as stock solutions (0.36 mM) in PBS and diluted into media. Only PBS was added as solvent control. Cells were washed two times with PBS and then treated with Tz–biotin **10** [19] (1 mM) for 1–3 h at 37 °C. After two washes with PBS, cells were incubated with Streptavidin–AF647 (6.6 μg mL^−1^) and Hoechst33342 (10 µg mL^−1^) for 20 minutes at room temperature in the dark. Cells were washed twice with PBS, and DMEM was added for microscopy. A Zeiss LSM 510 Meta equipped with a 40 × 1.3 NA Plan-Neofluar oil DIC immersion objective was employed for imaging. Analysis of the obtained data was performed using Image J software version 1.45 S.2.

**Western blot analysis.** HeLa S3 cells were seeded (900,000 cells/10 cm dish) and after 16 h the media were exchanged with media containing 100 µM of cyclopropene-labeled sugar (Ac_4_GlcNCyoc (**1**), Ac_4_GalNCyoc (**2**), or Ac_4_ManNCyoc (**3**)). Sugars were diluted from a 0.36 mM stock solution in PBS. As a solvent control PBS was added instead of the sugar stock solution. The cells were cultured for 48 h with or without the additional sugar. Cells were trypsinated and re-suspended in PBS (10 mL) and pelleted by centrifugation (5 min, 400*g*). The supernatant was discarded and the pellet re-suspended in PBS (1 mL) and pelleted by centrifugation (5 min, 400*g*). The cells were lysed in lysis buffer (400 µL) containing Triton X-100 (0.5%) (for permeabilization of membranes and solubilization of proteins and to prevent aggregate formation), DNase (30 µg mL^−1^), RNase (30 µg mL^−1^), β-glycerophosphate (20 mM) (Ser/Thr phosphatase inhibitor), sodium fluoride (20 mM) (Ser/Thr phosphatase inhibitor), sodium orthovandadate (0.3 mM) (inhibitor for ATPase, tyrosine and alkaline phosphatases), complete X protease inhibitor (Roche) (1X), NaCl (300 mM), TrisHCl pH 7.4 (25 mM), EDTA (5 mM) (to chelate metal ions and reduce oxidation damage), *O*-(2-acetamido-2-deoxy-D-glucopyranosylidenamino) *N*-phenylcarbamate (PUGNAc) (Sigma-Aldrich) (100 µM) (O-GlcNAc-β-*N*-acetylglucosaminidase inhibitor to maintain O-GlcNAcylation during lysis) and incubated at 4 °C for 30 min. The lysate was cleared by centrifugation (22,000*g*, 30 min, rt). Tz–biotin **10** was added to the cleared supernatant to a final concentration of 150 µM. The samples were incubated for 90 min at rt, 3× SDS-sample buffer was added, and the sample was boiled at 90 °C for 15 min. Proteins were separated by SDS-polyacrylamide gel electrophoresis using 10% polyacrylamide gels and transferred to nitrocellulose membranes (BioRad). Transfer efficiency was analyzed with Ponceau S staining. The membranes were blocked in milk (5% in PBS-T) for 1 h at rt, followed by incubation with anti-biotin antibody (Abnova, Anti-Biotin mAb clone SB58c, 1:2000 dilution in milk) at 4 °C overnight or anti-alpha-tubulin antibody (AA4.3, hypridoma supernatant in 1% FCS, 1:200 dilution in milk) for 1 h at rt. The membranes were washed (3 times, 10–15 min, PBS-T), incubated with secondary horseradish-peroxidase-conjugated anti-mouse antibody (Dianova, goat anti-mouse IgG (H+L)-HRP, 1:50000 dilution in milk, 1 h, rt), and washed again (3 times, 10–15 min, PBS-T). Blots were developed by an ECL detection system (clarity Western ECL substrate, BioRad) and visualised with a CCD camera (Raytest-1000, Fujifilm).

## Supporting Information

Additional MOE experiments and ^1^H and ^13^C NMR spectra of Ac_4_GlcNCyoc (**1**) and Ac_4_GalNCyoc (**2**).

File 1 Additional MOE experiments and NMR spectra.
