# Supplementary material for: Expanding the scope of cyclopropene reporters for the detection of metabolically engineered glycoproteins by Diels–Alder reactions
Source: Beilstein J Org Chem. 2014 Sep 22;10:2235–42. doi: 10.3762/bjoc.10.232 (PMC4187077; doi:10.3762/bjoc.10.232)
Supplement: File 1 — Additional MOE experiments and NMR spectra. [file Beilstein_J_Org_Chem-10-2235-s001.pdf]

Supporting Information

for

Expanding the scope of cyclopropene reporters for the detection of metabolically engineered glycoproteins by Diels–Alder reactions

Anne-Katrin Späte[1], Verena F. Schart[1], Julia Häfner[2], Andrea Niederwieser[1], Thomas U. Mayer[2], and Valentin Wittmann*,§,1

Address: [1]University of Konstanz, Department of Chemistry and Konstanz Research School Chemical Biology (KoRS-CB), Universitätsstraße 10, 78457 Konstanz (Germany) and [2]University of Konstanz, Department of Biology and Konstanz Research School Chemical Biology (KoRS-CB), Universitätsstraße 10, 78457 Konstanz (Germany)

Valentin Wittmann* - mail@valentin-wittmann.de

§Phone: +49-7531-884572, Fax: +49-7531-884573

*Corresponding Author

Additional MOE experiments and NMR spectra

Content

Additional MOE experiments S2

^{1}H and ^{13}C NMR spectra of Ac_4GlcNCyoc (**1**) and Ac_4GalNCyoc (**2**) S5

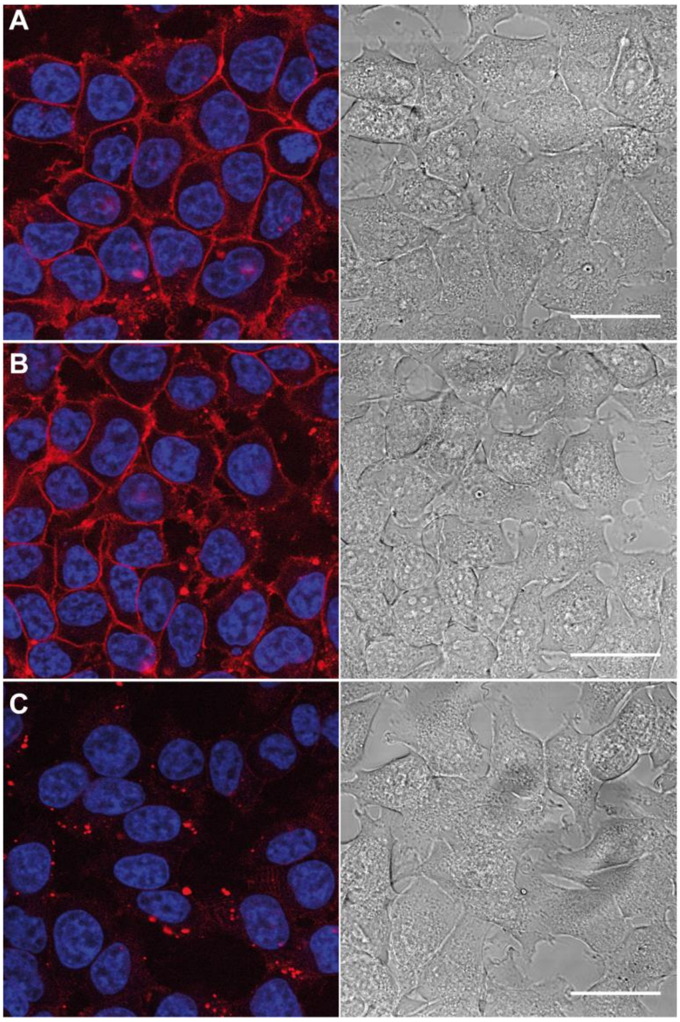

Figure S1: Labeling of metabolically engineered cell-surface glycoconjugates. HEK 293T cells were grown for 48 h with 50 µM Ac_4GlcNCyoc (**1**, A), 50 µM Ac_4GalNCyoc (**2**, B), or with PBS (solvent control, C) and subsequently incubated with Tz–biotin **10** (1 mM, 3 h, 37 °C) followed by incubation with streptavidin–AF647. Nuclei were stained with Hoechst33342. Scale bar: 30 µm.

Metabolic engineering with HeLa S3 cells

HeLa S3 cells (16,000 cells cm^{-2}) were seeded in 4-well ibiTreat µ-Slides (ibidi). After 12 h cells were incubated for 48 h with 50 µM cyclopropene-labeled sugar (Ac_4GlcNCyoc (**1**), Ac_4GalNCyoc (**2**) or Ac_4ManNCyoc (**3**)). The sugars were prepared as stock solutions in DMSO (100 mM) and diluted into media. DMSO only or 50 µM 1,3,4,6-tetra-*O*-acetyl-*N*-acetylglucosamine (Ac_4GlcNAc) were added as controls. Cells were washed two times with PBS and then treated with Tz–biotin **10** (1 mM) for 1 h at 37 °C. After two washes with PBS, cells were incubated with streptavidin–AlexaFluor555 (6.6 µg mL^{-1}) and Hoechst 33342 (10 µg mL^{-1}) for 20 min at 37 °C in the dark. Cells were washed twice with PBS, and DMEM was added for microscopy. A *Zeiss* LSM 780 equipped with a 40 x 1.4 Plan Apo oil DIC immersion objective and a GaAsP-detector array for spectral imaging was employed. Analysis of the obtained data was performed using Image J software version 1.45 S.2. Results are shown in Figure S2.

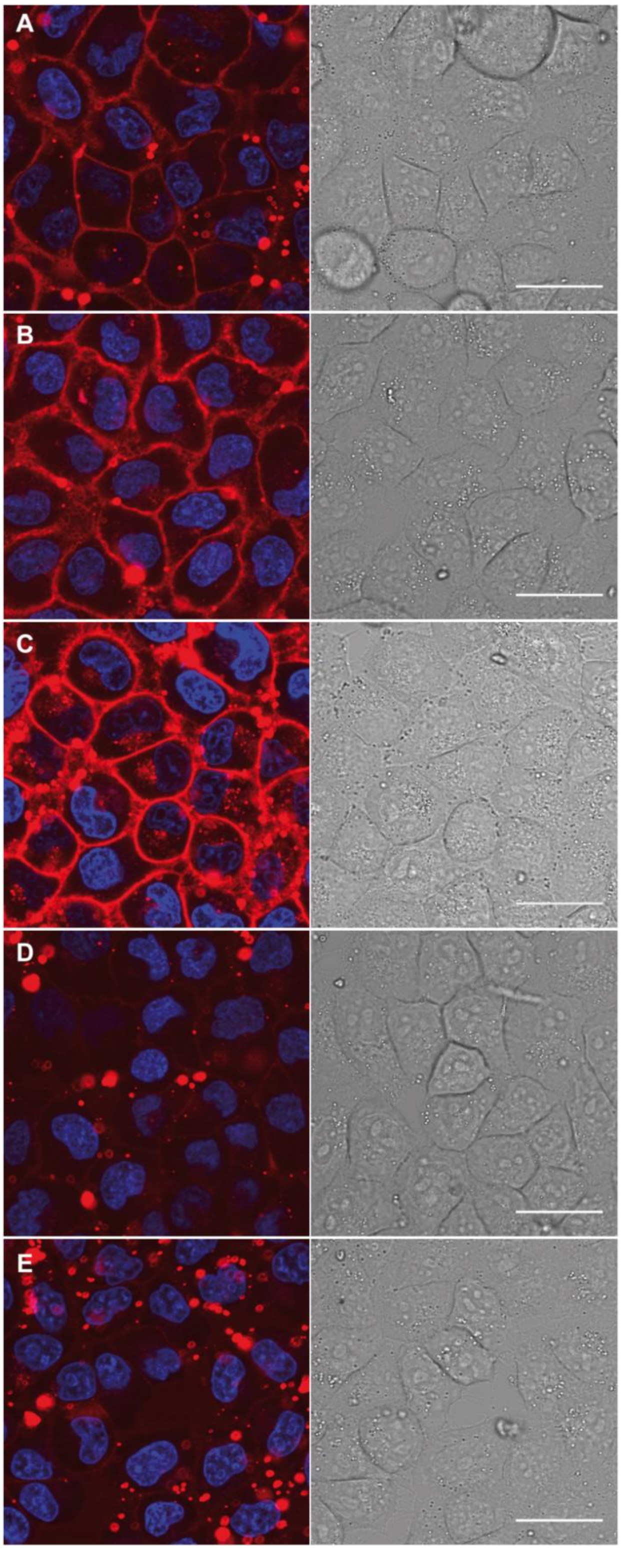

Figure S2: Labeling of metabolically engineered cell-surface glycoconjugates. HeLa S3 cells were grown for 48 h with 50 µM Ac_4GlcNCyoc (**1**, A), 50 µM Ac_4GalNCyoc (**2**, B), 50 µM Ac_4ManNCyoc (**3**, C), 50 µM Ac_4GlcNAc (D) or with DMSO (solvent control, E) and subsequently incubated with Tz–biotin **10** (1 mM, 1 h, 37 °C) followed by incubation with streptavidin–AF555. Nuclei were stained with Hoechst33342. Scale bar: 30 µm.

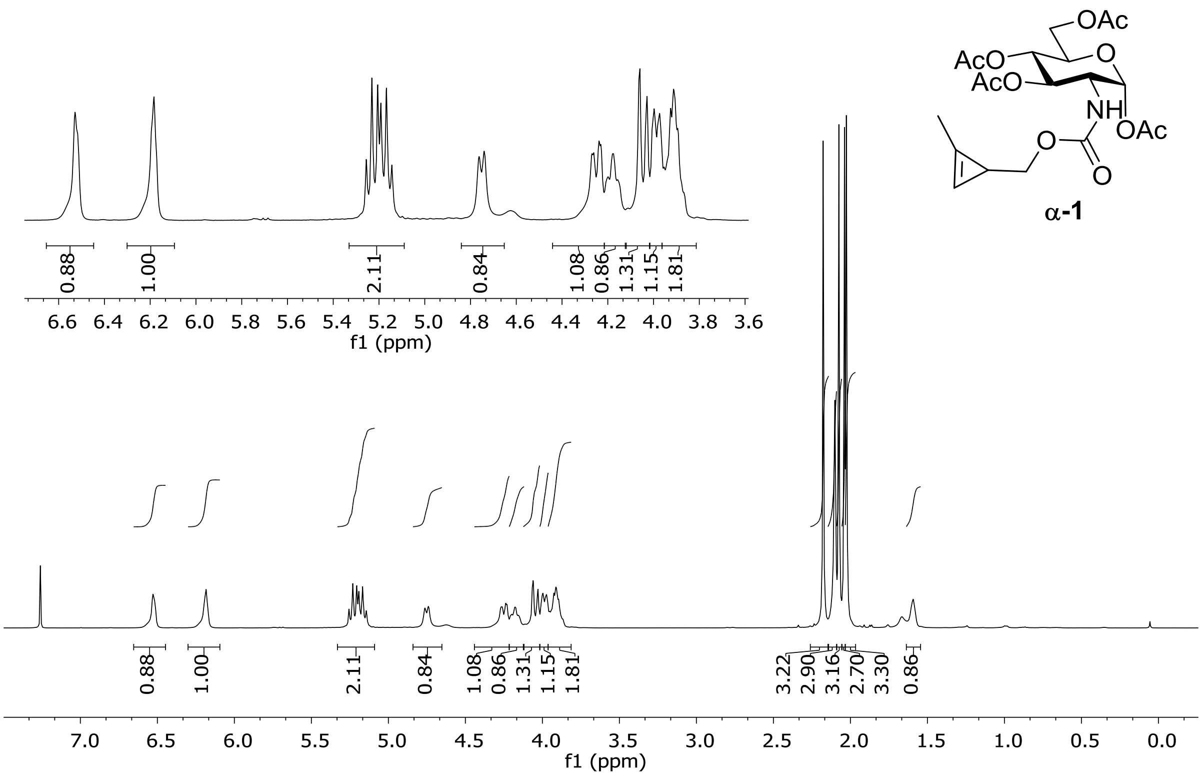

^{1}H NMR (400 MHz, $CDCl_3$)

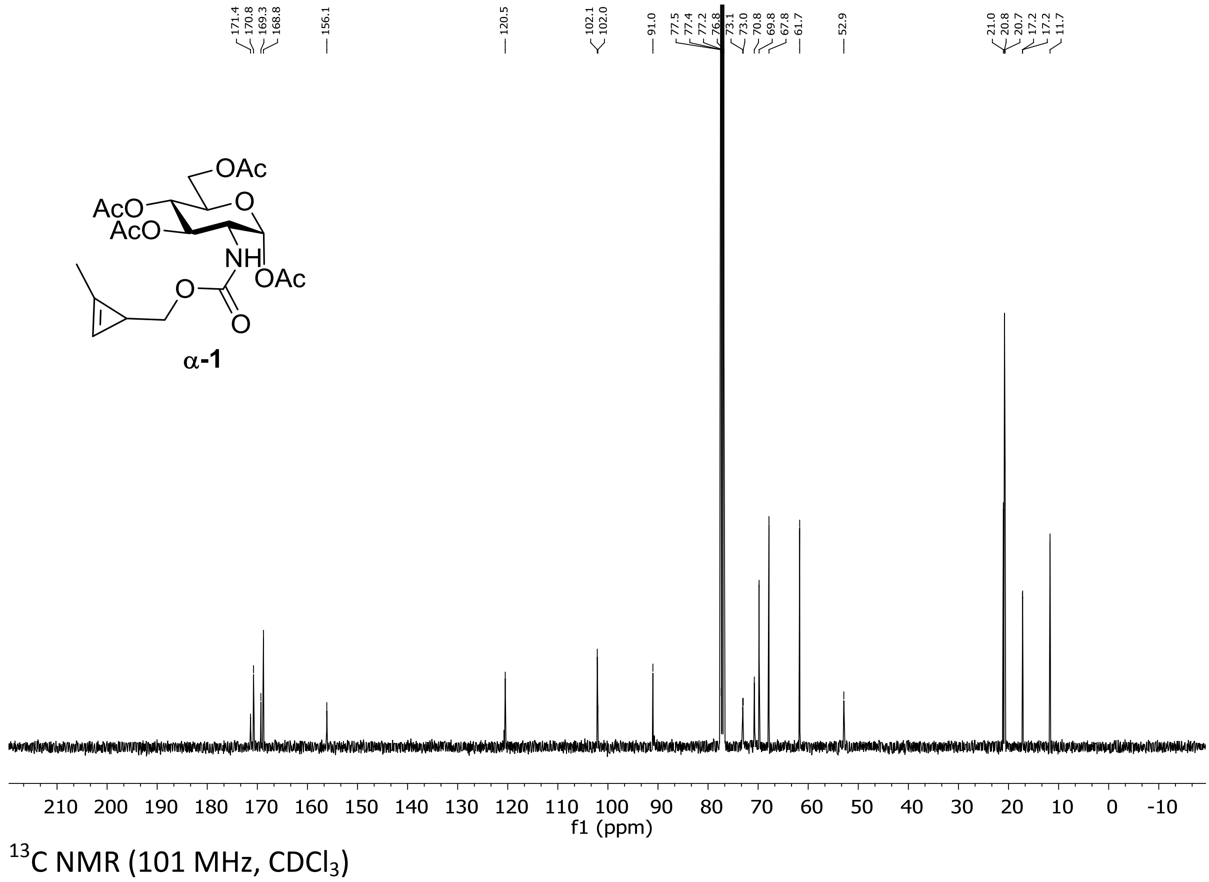

^{13}C NMR (101 MHz, $CDCl_3$)

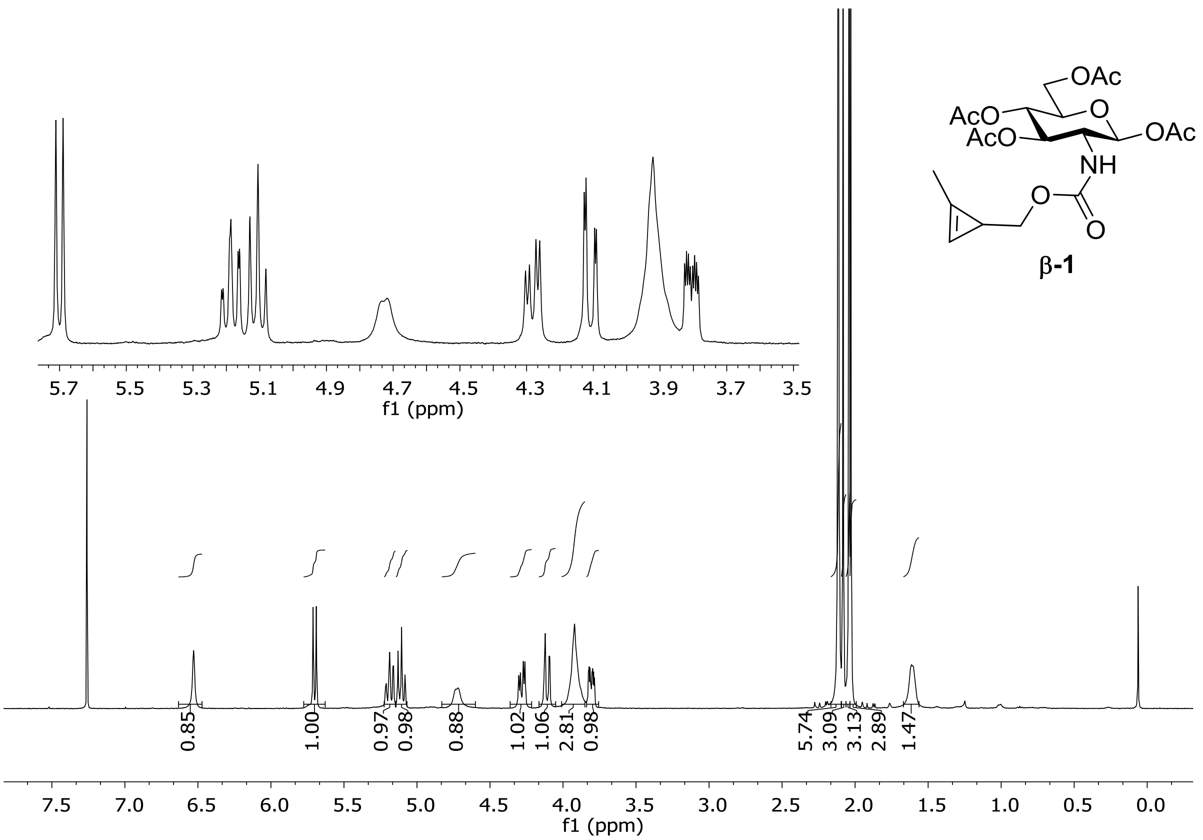

^{1}H NMR (400 MHz, $CDCl_3$)

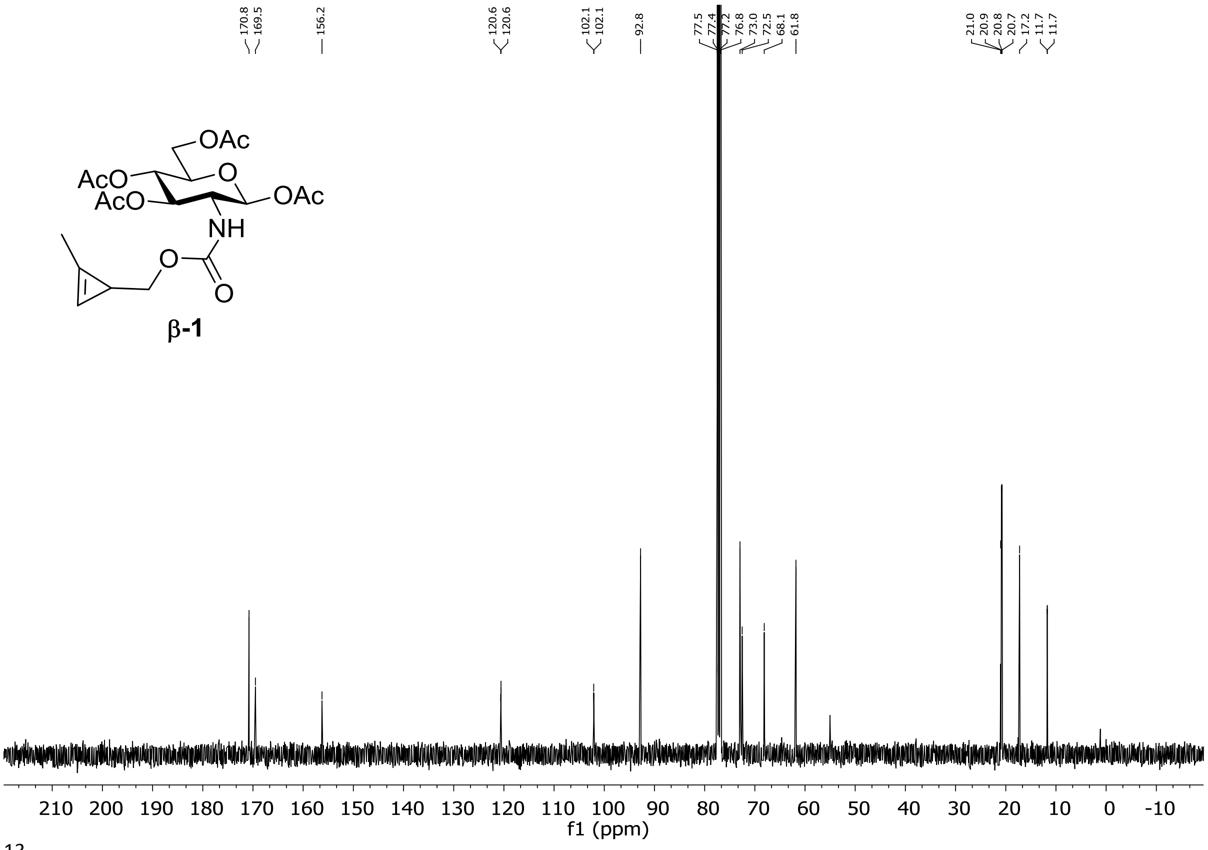

^{13}C NMR (101 MHz, $CDCl_3$)

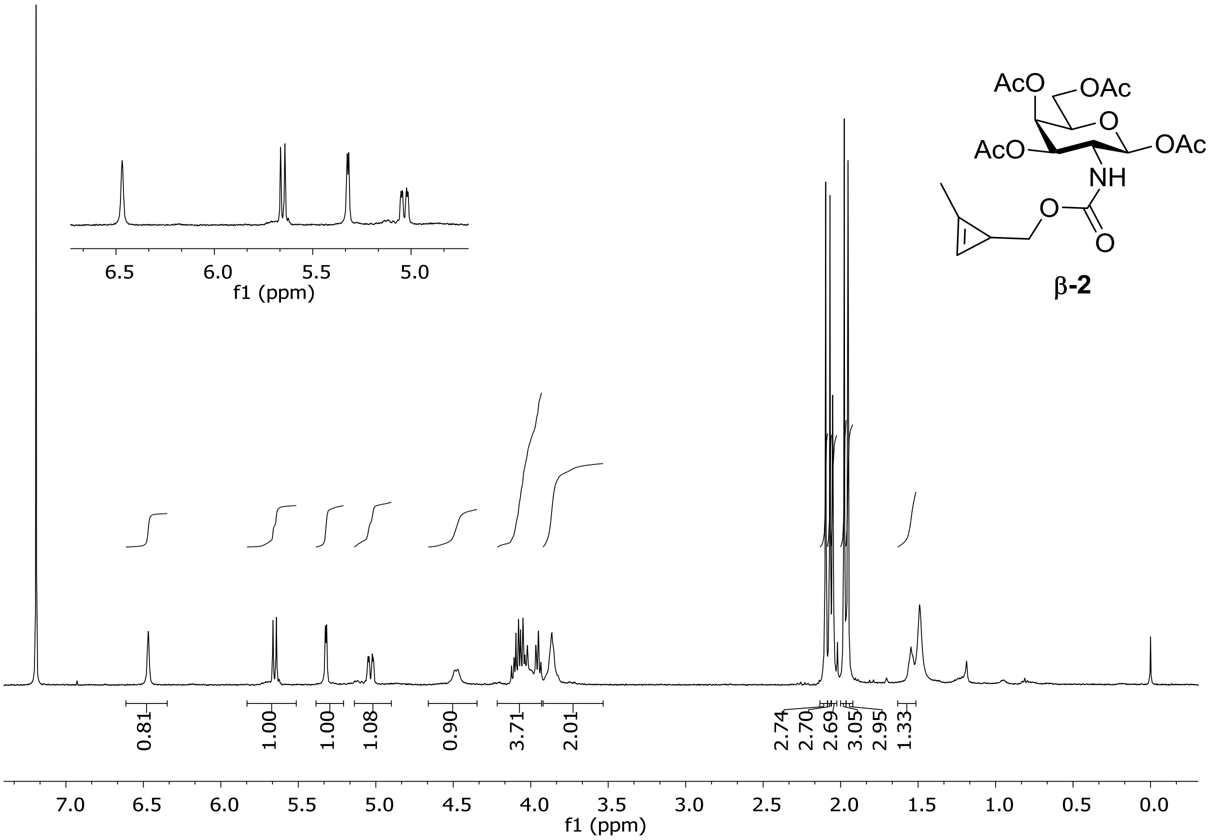

^{1}H NMR (400 MHz, $CDCl_3$)

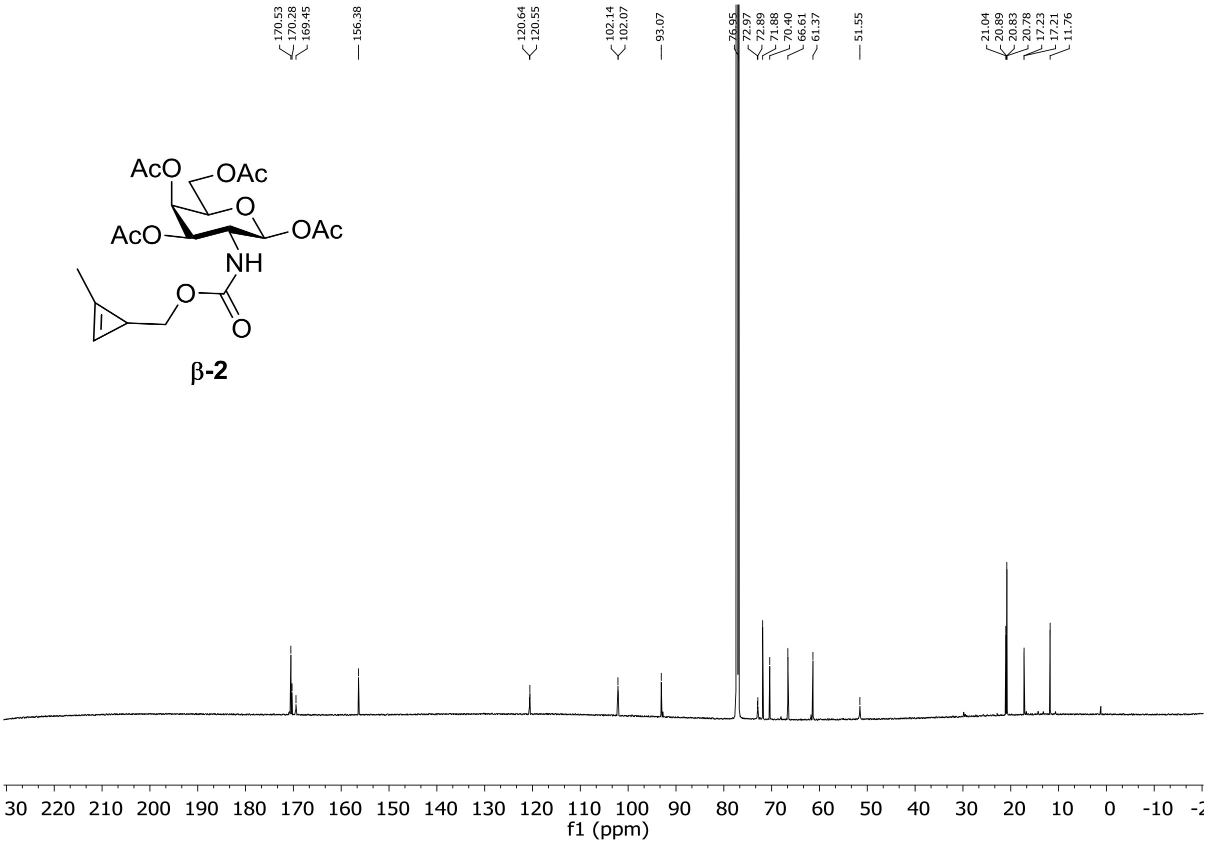

^{13}C NMR (101 MHz, $CDCl_3$)
